# TiO_2_ Nanosheet Arrays with Layered SnS_2_ and CoO_x_ Nanoparticles for Efficient Photoelectrochemical Water Splitting

**DOI:** 10.1186/s11671-019-3168-7

**Published:** 2019-11-11

**Authors:** Zhou Cao, Yanling Yin, Peng Fu, Dong Li, Yulan Zhou, Yuanwen Deng, Yuehua Peng, Weike Wang, Weichang Zhou, Dongsheng Tang

**Affiliations:** Synergetic Innovation Center for Quantum Effects and Application, Key Laboratory of Low-dimensional Quantum Structures and Quantum Control of Ministry of Education, School of Physics and Electronics, Hunan Normal University, Changsha, 410081 People’s Republic of China

**Keywords:** TiO_2_ nanosheet arrays, Tin sulfide, Heterojunction, Photocatalysis, Photoelectrochemical water splitting

## Abstract

Converting solar energy into sustainable hydrogen fuel by photoelectrochemical (PEC) water splitting is a promising technology to solve increasingly serious global energy supply and environmental issues. However, the PEC performance based on TiO_2_ nanomaterials is hindered by the limited sunlight-harvesting ability and its high recombination rate of photogenerated charge carriers. In this work, layered SnS_2_ absorbers and CoO_x_ nanoparticles decorated two-dimensional (2D) TiO_2_ nanosheet array photoelectrode have been rationally designed and successfully synthesized, which remarkably enhanced the PEC performance for water splitting. As the result, photoconversion efficiency of TiO_2_/SnS_2_/CoO_x_ and TiO_2_/SnS_2_ hybrid photoanodes increases by 3.6 and 2.0 times under simulated sunlight illumination, compared with the bare TiO_2_ nanosheet arrays photoanode. Furthermore, the TiO_2_/SnS_2_/CoO_x_ photoanode also presented higher PEC stability owing to CoO_x_ catalyst served as efficient water oxidation catalyst as well as an effective protectant for preventing absorber photocorrosion.

## Background

Nowadays, with the emergence of non-renewable fossil fuel concerns and environmental pollutions caused by conventional fossil fuel combustion, there is an urgent need to seek a sustainable clean, high photostability, non-toxicity, low cost, and environmental strategy for the generation of clean fuels [1–4]. Photoelectrochemical (PEC) water splitting is well recognized as an ideal alternative to explore attractive sustainable energy sources and technologies since the initial report on PEC water splitting in 1972 [5–7]. Photogenerated electron-hole pairs are spatially separated and transferred and subsequently participates in the water-splitting processes. Titanium dioxide (TiO_2_) is a promising semiconductor material candidate owing to its intrinsic advantages of high chemical stability, favorable band edge positions, earth-abundant, and nontoxicity [8–11]. However, TiO_2_, as a large band-gap semiconductor (ca. 3.2 eV), only absorb the ultraviolet (UV) light. Moreover, its high rate of photoinduced charge carrier recombination and low photoelectric-conversion efficiency limit substantially the practical photocatalytic activity [12–15]. It is highly desirable to construct the efficient geometric nanostructures for improving photoconversion efficiency of PEC water splitting, such as nanowires [16], nanorods [17], nanotubes [18], nanobelts [19], and nanofibers [20]. Recently, different morphological TiO_2_ materials have been applied to drive water splitting by using sunlight [21–23]. However, the water-splitting efficiency is unsatisfactory owing to the accompaniment of grain boundary effect and deficient specific surface area in these nanostructures. Therefore, two-dimensional (2D) vertically aligned TiO_2_ nanosheet array structure has attracted intense interest in the PEC water splitting. Compared to other one-dimensional (1D) nanostructures, anatase TiO_2_ nanosheet arrays with high proportion of exposed {001} facets have been proven to be an active phase when used as a photocatalyst [24–27]. Besides, the vertically grown TiO_2_ nanosheet arrays provide an unobstructed transportation pathway for electron transfer to substrates, and the high photocatalytic activity {001} facet-dominated anatase TiO_2_ has an extraordinary advantage on the separation of photogenerated charge carriers.

Nevertheless, the practical applications of TiO_2_-based water splitting systems are limited because not only the narrow light absorption region resulted from large bandgap, but also its low quantum efficiency and high photogenerated charge carriers recombination rate. Therefore, considerable efforts have been paid to improve the solar light absorption ability and conversion efficiency, for instance, ions doping [28, 29], coupling metal plasmonic nanostructures [30–32], or photosensitization of semiconductors with small bandgap [33–35]. Alternatively, heterogeneous junction constructed with narrow-bandgap photosensitizer has been widely recognized to be an available method to promote efficiently charge carrier separation and extend light absorption ability of the photocatalytic materials [36–39]. Typically, tin (IV) disulfide (SnS_2_) with a suitable bandgap energy of 2.4 eV has attracted significant attention for its remarkable optical and electrical properties. As a member of the layered metal chalcogenide semiconductor, 2D SnS_2_ nanosheets have been demonstrated as an attractive photocatalyst in PEC solar water splitting because of the effective light absorption ability, short carrier transport distances, and large specific surface area [40–43]. Alternatively, the type II heterojunction combined SnS_2_ with TiO_2_ has been considered as an efficient route to enhance significantly the light absorption ability as well as improve charge separation efficiency [44, 45]. Furthermore, oxygen evolution, which is the four electrons transfer reaction, is usually considered to be a kinetics controlling step. The water-splitting efficiency can be further enhanced through the integration of cobalt-based catalysts; the catalyst acts as active sites for water oxidation, provides a lower over-potential, and prevents photocorrosion in the water-splitting process [46–48].

In this work, vertically aligned TiO_2_ nanosheet arrays were applied in TiO_2_/SnS_2_/CoO_x_ heterojunction photoelectrodes for PEC water splitting. CoO_x_ nanoparticles, which are known to be excellent water oxidation catalysts, were loaded on TiO_2_/SnS_2_ nanosheet arrays to construct triple hybrid photoanodes. The hybrid semiconducting photoanodes were fabricated by simple hydrothermal or solvothermal process, and the detailed prepared method characterization was discussed subsequently. With CoO_x_ loading, the performance of TiO_2_/SnS_2_ photoanode was improved markedly. TiO_2_/SnS_2_/CoO_x_ composite nanosheet array photoanode exhibits remarkably improved performances for the PEC water splitting.

## Methods

### Chemicals and Reagents

Tetrabutyl titanate (C_16_H_36_O_4_Ti, Aladdin Chemistry Co., Ltd., ≥ 99%), ammonium hexafluorotitanate ((NH_4_)_2_TiF_6_, Sinopharm Chemical Reagent Co., Ltd., AR), tin (IV) chloride pentahydrate (SnCl_4_·5H_2_O, Sinopharm Chemical Reagent Co., Ltd., ≥ 99%), thioacetamide (CH_3_CSNH_2_, Sinopharm Chemical Reagent Co., Ltd., ≥ 99%), cobalt (II) acetate tetrahydrate (Co (CH_3_COO)_2_·4H_2_O, Sinopharm Chemical Reagent Co., Ltd., ≥ 99.5%), ammonium solution (NH_3_·H_2_O, 25 wt%), concentrated hydrochloric acid (36–38 wt%), acetone (AR), and ethanol (AR) were obtained from Tianjin Chemical Reagents Plant, China. All chemicals were used as received without any further purification.

### Preparation of TiO_2_ Nanosheet Arrays

TiO_2_ nanosheet array photoelectrodes were fabricated onto fluorine-doped tin oxide (FTO)-coated conductive glass substrates using a facile hydrothermal process [49]. In a typical procedure, 10 ml of concentrated hydrochloric acid and 10 ml of deionized (DI) water (18.25 MΩ cm) were mixed under strong stirring at room temperature. Subsequently, 0.4 ml of tetrabutyl titanate was dropped to the mixed solution and stirred vigorously for 5 min to obtain a transparent solution. Next, 0.2 g of ammonium hexafluorotitanate ((NH_4_)_2_TiF_6_) was added and further stirred for 10 min. The as-prepared mixture precursor solution was transferred to a Teflon-lined autoclave (100 ml in volume). The FTO substrates (14 Ω/square) were ultrasonically cleaned with acetone, ethanol, and DI water in sequence and dried prior to the experiment. Then, the conductive FTO substrate was placed facing down into the autoclave obliquely. The autoclave was conducted at 170 °C for 10 h and then naturally cooled down. After the synthesis, the sample was washed with DI water and air-dried at room temperature. To increase the crystallinity of TiO_2_ nanosheet arrays, the as-prepared samples were annealed in air atmosphere at 550 °C for 3 h.

### Fabrication of TiO_2_/SnS_2_ Hybrid

The hybrid TiO_2_/SnS_2_ nanosheet arrays can be fabricated as described in the following preparation details; 2D SnS_2_ were grown on TiO_2_ nanosheet arrays by low-temperature solvothermal method. A mixture solution containing 10 ml absolute ethanol, 10 mM SnCl_4_, and 30 mM thioacetamide was magnetically stirred and prepared in the solvothermal process. Then FTO substrates covered with TiO_2_ nanosheet arrays were vertically inserted into the precursor solution. During the deposition, the temperature was heated at 80 °C for 1 h. After cooling down, the fabricated samples were rinsed by absolute ethanol and DI water several times and annealed in Ar atmosphere at 250 °C for 2 h.

### Synthesis of TiO_2_/SnS_2_/CoO_x_ Photoelectrodes

Finally, CoO_x_ nanoparticles were loaded on TiO_2_/SnS_2_ nanosheet arrays by a modified solvothermal method reported previously [50, 51]. In detail, 0.25 ml ammonium solution was dropwise added into 18 ml ethanol solution containing 5 mM cobalt acetate under vigorous stirring. Subsequently, the as-prepared solution was transferred into a 25-ml autoclave and two pieces of TiO_2_/SnS_2_ electrodes were obliquely placed into the bottom of the autoclave. Next, the autoclave was heated and kept at 120 °C for 1 h. After the solvothermal process finished, the obtained TiO_2_/SnS_2_/CoO_x_ photoelectrodes were thoroughly rinsed with DI water and dried in air.

### Characterization

X-ray diffraction (XRD) patterns were obtained using a Bruker D8 Discover X-ray diffractometer with Cu Kα radiation (*λ* = 0.15406 nm). Scanning electron microscopic images were obtained using a FEI NovaSEM-450 field emission scanning electron microscope (SEM) equipped with an Oxford X-max20 energy dispersive X-ray spectrometer (EDS). The optical absorption spectra were recorded on a Perkin Elmer Lambda 750 coupled with a 60-mm integrating sphere attachment. Transmission electron microscopy (TEM) images were recorded in a FEI Tecnai F20 transmission electron microscope with operating voltage 200 kV. Raman spectra were recorded on a LabRAM HR Evolution Horiba JY high-resolution Raman spectrometer with a wavelength of 633 nm as the excitation source. X-ray photoelectron spectroscopy (XPS) was recorded by a Thermo Fisher Scientific-Escalab 250Xi X-ray photoelectron spectrometer with a monochromatic Al Ka irradiation.

### PEC Measurements

PEC measurements were carried out using a standard three-electrode cell with the fabricated electrode used as a working electrode, a Pt wire used as a counter electrode, and Ag/AgCl used as reference electrode at an electrochemical workstation (CorrTest, CS350). All PEC measurements were performed with the effective surface area of the working electrode kept as 2 cm^2^ and illuminated from the front side in 0.5 M Na_2_SO_4_ (pH = 6.8) electrolyte. The electrode potential of the working electrodes (vs. Ag/AgCl) can be converted to the reversible hydrogen electrode (RHE) potential by the Nernst equations: ${E}_{\mathrm{RHE}}={E}_{\mathrm{Ag}/\mathrm{AgCl}}+0.059\ \mathrm{pH}+{E}_{\mathrm{Ag}/\mathrm{AgCl}}^{\uptheta}$, where *E*_RHE_ is the converted potential vs. RHE, ${E}_{\mathrm{Ag}/\mathrm{AgCl}}^{\uptheta}$ is 0.1976 V at 25 °C, and *E*_Ag/AgCl_ is the applied potential against the Ag/AgCl reference electrode. The photocurrent density-potential (*i*-*v*) measurements were carried out at a scan rate of 10 mV/s under the solar simulator (7IS0503A) using a 150 W xenon lamp equipped with an AM 1.5G filter as illumination source (100 mW/cm^2^). The amperometric photocurrent-time (*i*-*t*) curves were evaluated with light irradiation on/off cycles under an applied potential of 1.23 V vs. RHE. Electrochemical impedance spectroscopy (EIS) was carried out in the frequency range of 0.01–100 kHz and an AC voltage amplitude of 5 mV at an open-circuit potential.

## Results and Discussion

**Fig. 1:**
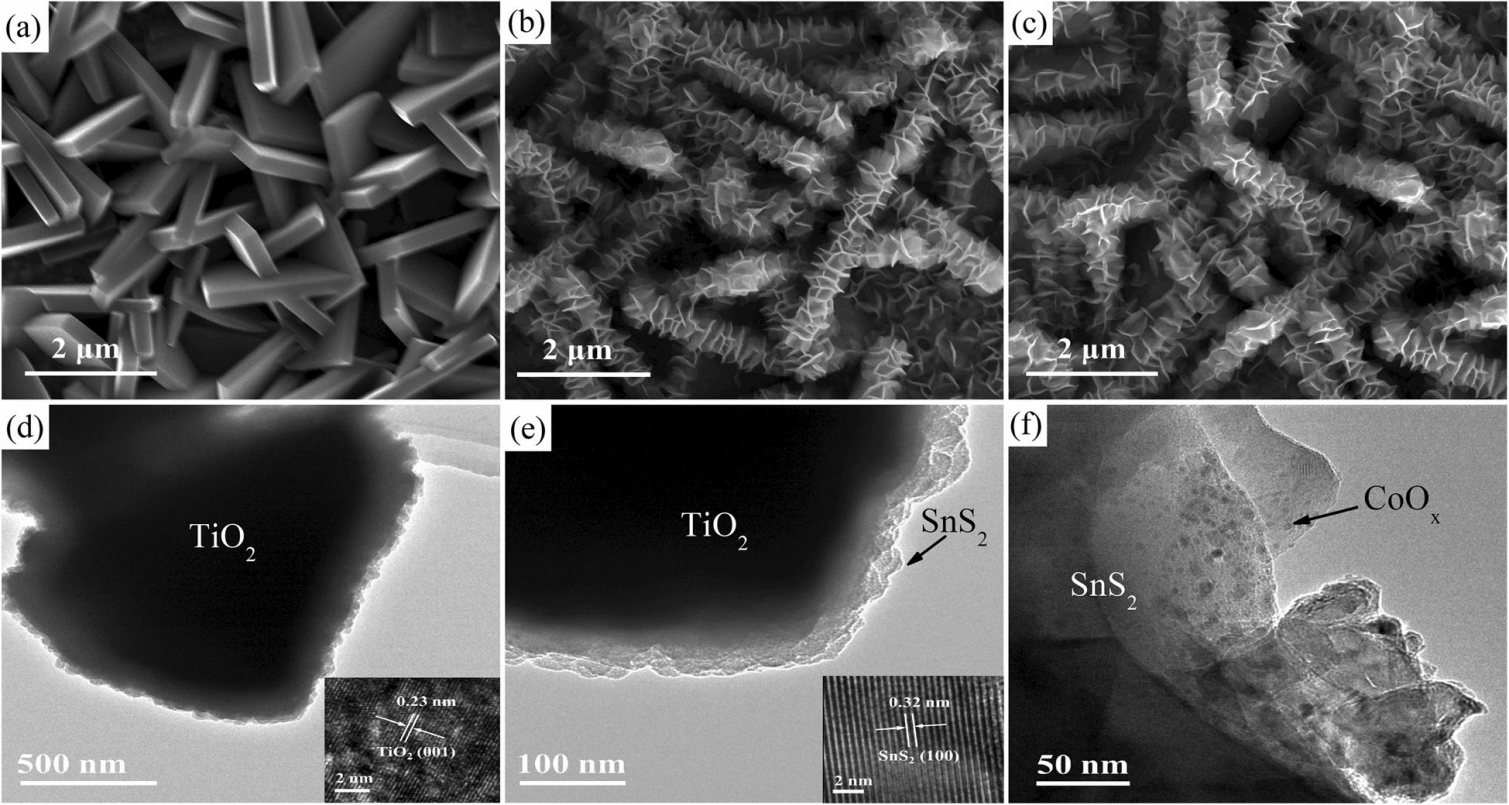
SEM images of **a** TiO_2_ nanosheet arrays, **b** TiO_2_/SnS_2_ nanosheet arrays, and **c** TiO_2_/SnS_2_/CoO_x_ nanosheet arrays. **d–f** TEM images of TiO_2_/SnS_2_/CoO_x_ nanosheet arrays. The insets of **d** and **e** show the HRTEM images of TiO_2_ and SnS_2_, respectively

The process for fabrication of the TiO_2_/SnS_2_/CoO_x_ nanosheet array photoanode is illustrated (Additional file 1: Scheme S1). The morphology and structure images of the pristine TiO_2_ and hybrid nanosheet array photoelectrodes are displayed in Fig. 1 by SEM and TEM observation. In order to ensure that each photoelectrode has an equal density of nanosheet arrays, the pristine TiO_2_ nanosheet array photoelectrode was prepared in one-pot hydrothermal synthesis. Obviously, the surface of FTO substrate is uniformly covered with smooth TiO_2_ nanosheet arrays and the thickness of nanosheet is typically about 280 nm as observed from Fig. 1a. In addition, the cross-section image shows that the film is composed of vertically aligned TiO_2_ nanosheet arrays and the height of nanosheet arrays is about 1 μm (Additional file 1: Figure S1). It is apparent that the entire surfaces of TiO_2_ nanosheet arrays become rough after the deposition of SnS_2_ layer (Fig. 1b). With the loading of CoO_x_ nanoparticles, the SEM picture of the nanosheet arrays has almost no significant difference owing to CoO_x_ nanoparticle high dispersion and low concentration, as shown in Fig. 1c. However, EDS reflect the presence of CoO_x_ nanoparticles on the surface of hybrid (Additional file 1: Figure S2). As revealed by Fig. 1d, HRTEM images further reveal that the nanosheets have a single-crystalline structure, which clearly shows the lattice fringes of 0.23 nm, corresponding to the *d*-spacing values of the anatase TiO_2_ (001) planes. In the TEM image in Fig. 1e of an individual TiO_2_/SnS_2_ heterojunction nanosheet, it clearly illustrates that the TiO_2_ nanosheets are covered by the SnS_2_ outlayer. As can be seen in the HRTEM images, the lattice *d*-spacing is 0.32 nm, corresponding to (100) fringe plane of hexagonal SnS_2_. As seen in Fig. 1f, the HRTEM image shows that CoO_x_ nanoparticles are evenly dispersed on the surface of TiO_2_/SnS_2_ nanosheet arrays.

**Fig. 2:**
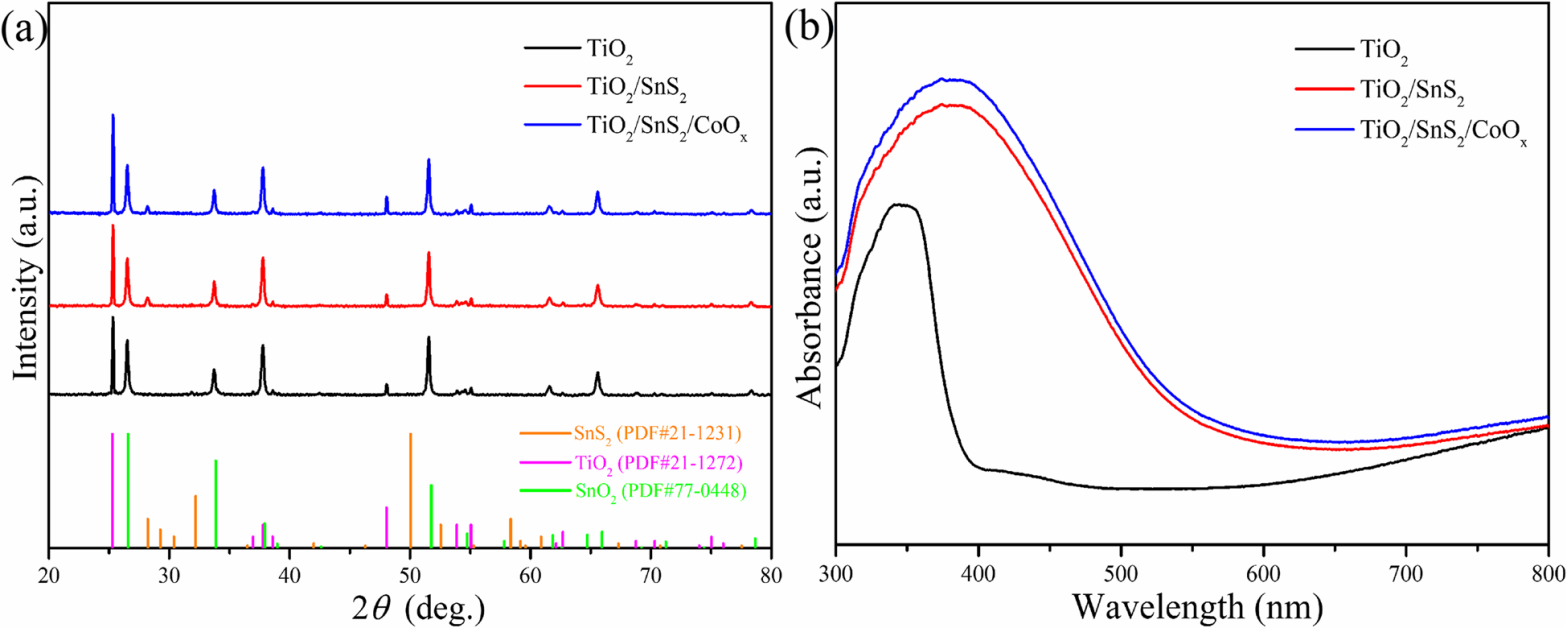
**a** XRD pattern and **b** absorption spectra of pristine TiO_2_, TiO_2_/SnS_2_ and TiO_2_/SnS_2_/CoO_x_ nanosheet arrays

XRD measurement was used to identify the crystallinity and crystal structure of hybrid photoelectrodes. As described in Fig. 2a, all of the diffraction peaks are readily indexed to the typical anatase TiO_2_ (JCPDS 21-1272) and hexagonal SnS_2_ (JCPDS 21-1231) apart from the FTO substrate peaks, which revealed the coexistence of TiO_2_ and SnS_2_ in the hybrid electrodes. However, diffraction peaks corresponding to CoO_x_ (CoO or Co_3_O_4_) were not evidently detected, probably because of their low concentration and high dispersion on the hybrid electrode surface. To further verify the crystalline phase of hybrid photoelectrodes, an additional Raman spectrum was performed (Additional file 1: Figure S3). The Raman spectrum for the TiO_2_ nanosheet arrays shows characteristic bands at around 144, 394, 514, and 637 cm^−l^, corresponding to the Raman active modes in anatase TiO_2_ with the O–Ti–O vibration of *E*_g_, *B*_1g_, *A*_1g_, and *E*_g_, respectively [52–54]. The same Raman scattering peaks are observed for the TiO_2_/SnS_2_ sample. After the formation of TiO_2_/SnS_2_ heterojunction, the *A*_1g_ mode Raman peak of hexagonal SnS_2_ at 314 cm^−1^ is observed, verifying the successful introduction of SnS_2_ layers in the hybrid electrode [55, 56]. The optical absorption spectra of bare TiO_2_, TiO_2_/SnS_2_, and TiO_2_/SnS_2_/CoO_x_ nanosheet arrays are presented in Fig. 2b. The pristine TiO_2_ nanosheet array sample shows the characteristic absorption band located at 380 nm, while the TiO_2_/SnS_2_ hybrid appears a wide visible light absorption edge, which was attributed to the excellent light absorption ability of SnS_2_ layer. The corresponding optical energy gap can be subsequently calculated using the following equation: *αhν* = *A*(*hν* − *E*_*g*_)^*n*^, where *α*, *A*, *hν*, and *E*_*g*_ are the optical absorption coefficient, a constant, incident photon energy, and the bandgap, respectively. In addition, *n* is equal to 1/2 for direct bandgap semiconductors while *n* is equal to 2 for indirect bandgap semiconductors. The energy gap for the bare TiO_2_ and pristine SnS_2_ was estimated to be 3.2 and 2.4 eV (Additional file 1: Figure S4), respectively [57–60]. After decorating with CoO_x_, the absorption spectra of TiO_2_/SnS_2_/CoO_x_ nanosheet arrays display similar light absorption bands (ca. 560 nm) to TiO_2_/SnS_2_ hybrid, which implies absent additional bandgap transition resulted from the introduction of CoO_x_ catalysts.

**Fig. 3:**
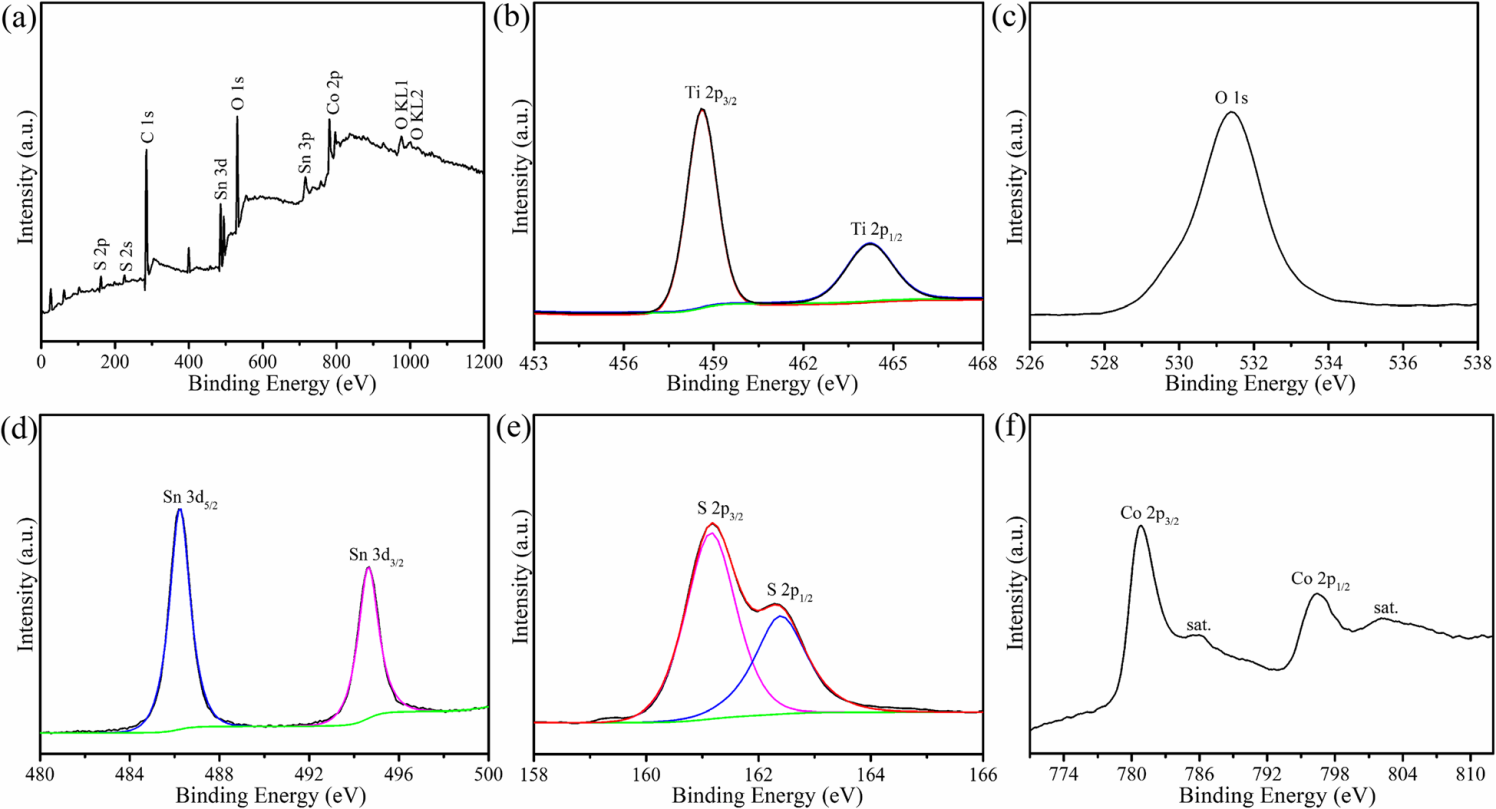
XPS survey spectra (**a**), high-resolution XPS spectra of **b** Ti 2p, **c** O 1s, **d** Sn 3d, **e** S 2p, and **f** Co 2p for TiO_2_/SnS_2_/CoO_x_ composite nanosheet arrays

To further investigate the valence state and chemical environment, XPS characterization of all the photoelectrodes was measured. As illustrated in Fig. 3a, XPS survey spectrum of the TiO_2_/SnS_2_/CoO_x_ hybrid proves the presence of Ti, O, Sn, S, and Co elements. Figure 3b shows the high-resolution XPS spectrum of Ti 2p. The two peaks located at 458.6 and 464.2 eV are ascribed to Ti 2p_3/2_ and Ti 2p_1/2_, respectively, indicating the presence of Ti^4+^ species. Figure 3c shows the binding energy of the O 1s core level around 531.4 eV, which is corresponding to the lattice oxygen atoms of Ti–O–Ti bond. Two symmetric peaks at the binding energy of 486.47 (Sn 3d_5/2_) and 494.88 eV (Sn 3d_3/2_) are shown in Fig. 3d, which confirmed the existence of Sn^4+^ in the hybrid electrodes. Meanwhile, the peaks located at 161.2 and 162.3 eV are corresponding to S 2p_3/2_ and S 2p_1/2_ states (Fig. 3e), demonstrating the formation of the SnS_2_ outlayer. Furthermore, two distinct peaks located at 796.5 (Co 2p_1/2_) and 780.6 eV (Co 2p_3/2_) with the satellite peaks are presented in Fig. 3f, which is ascribed to the coordination of both the Co^3+^ and Co^2+^. That is a demonstration, in fact, that the water oxidation catalyst CoO_x_ (CoO and Co_3_O_4_) is definitely assembled on the surface of hybrid photoelectrodes. In addition, the atomic percentage of Co element was estimated to be about 4.3 at% based on XPS analysis in the TiO_2_/SnS_2_/CoO_x_ nanosheet arrays. As a result, the diffraction peak performed on the previous XRD measurement is not detected because of the low concentration of CoO_x_ nanoparticles in the hybrid photoelectrodes.

**Fig. 4:**
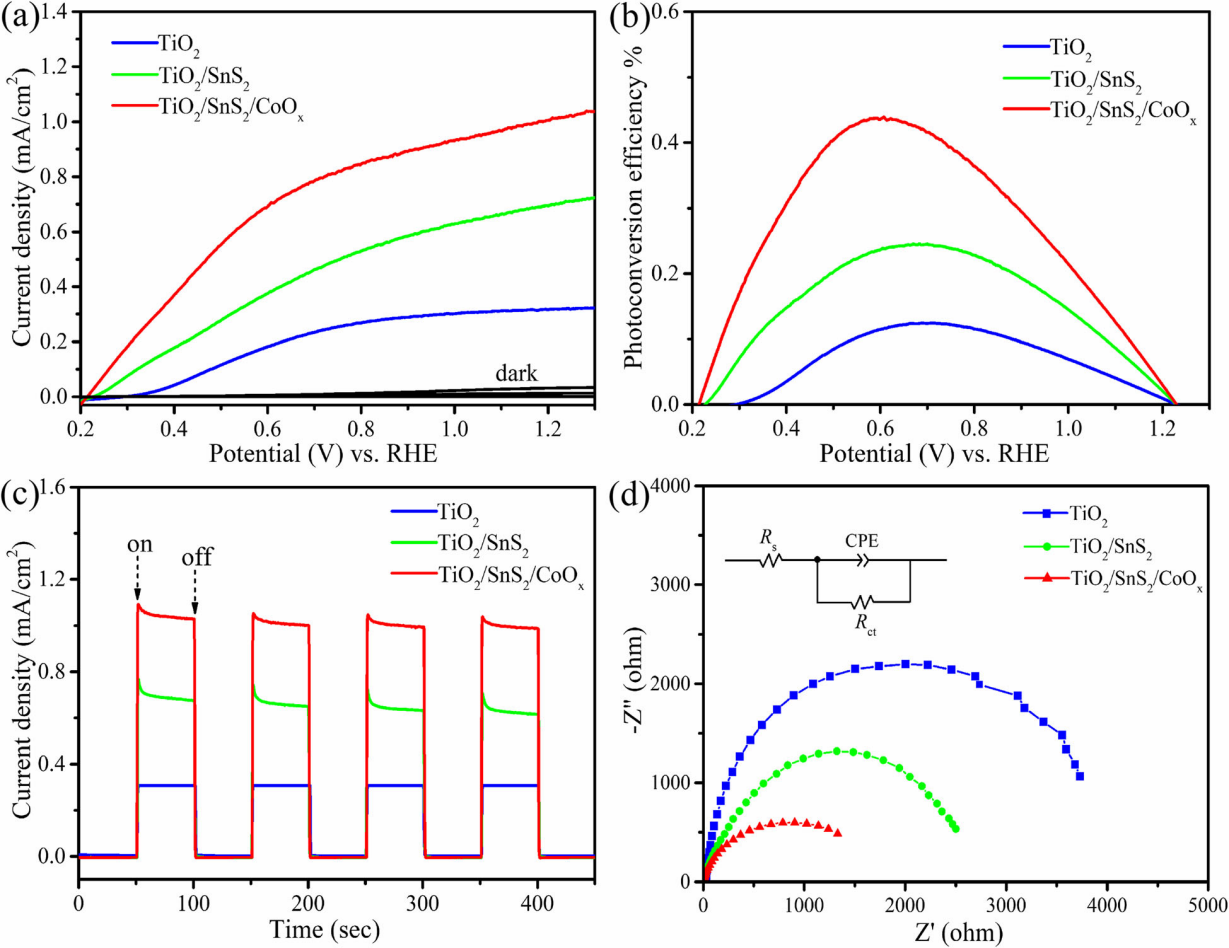
PEC measurements for pristine TiO_2_, TiO_2_/SnS_2_, and TiO_2_/SnS_2_/CoO_x_ photoelectrodes in 0.5 M Na_2_SO_4_ electrolyte. **a** Photocurrent density-applied potential characteristics. **b** Calculated photoconversion efficiencies. **c** Photocurrent density-time plots measured at 1.23 V vs. RHE under chopped light irradiation. **d** EIS spectra measured under irradiation

To investigate the PEC performance of these photoelectrodes, the nanosheet arrays were fabricated into the working electrode in a standard three-electrode electrochemical system. Linear sweep voltammetry (LSV) curves of the pristine TiO_2_, TiO_2_/SnS_2_, and TiO_2_/SnS_2_/CoO_x_ nanosheet array photoelectrodes are shown in Fig. 4a, in an applied potential range of 0.2 to 1.3 V vs. RHE. Obviously, dark scan LSV curves show an almost negligible current density for all samples. Nevertheless, the photocurrent of TiO_2_ electrode is remarkably enhanced after coated with SnS_2_ photosensitizer and then further improved when deposited CoO_x_ catalysts under simulated sunlight illumination. Furthermore, the onset potential of photocurrent for TiO_2_ nanosheet arrays is negatively shifted for TiO_2_/SnS_2_ and TiO_2_/SnS_2_/CoO_x_ nanosheet array electrodes, due to the negative shift of Fermi level and low carrier recombination rate by SnS_2_ outlayer and CoO_x_ catalysts. In addition, the photoconversion efficiency (*η*) of pristine TiO_2_ and TiO_2_/SnS_2_ and TiO_2_/SnS_2_/CoO_x_ photoelectrodes are calculated using the following equation:

$$ \eta =I\ \left({E}_{\mathrm{rev}}^{\uptheta}-V\right)/{J}_{\mathrm{light}} $$

where *I* is the photocurrent density (mA/cm^2^), *E*θ rev is 1.23 V vs. RHE for the water splitting, *V* is the measured potential vs. RHE, and *J*_light_ is the irradiance intensity of incident light (100 mW/cm^2^). Figure 4b displays the photoconversion efficiency plots with applied potential from 0.2 to 1.3 V vs. RHE under light radiation. The pristine TiO_2_ photoelectrode displays the optimal photoconversion efficiency of 0.12% at 0.70 V vs. RHE. Remarkably, TiO_2_/SnS_2_/CoO_x_ and TiO_2_/SnS_2_ nanosheet array photoelectrodes exhibit the highest efficiency of 0.44% and 0.24%, about 3.6 and 2.0 times higher compared with pristine TiO_2_ nanosheet arrays, respectively. The chopped light photoresponse (*i*-*t*) curves of the photoanodes measured at 1.23 V vs. RHE, as shown in Fig. 4c. The fast rise-fall changing of the photocurrent density indicates that the charge transport in the photoelectrodes is very quick. In contrast, TiO_2_/SnS_2_/CoO_x_ photoelectrode exhibits a higher photocurrent density of 1.05 mA/cm^2^, 3.38-fold enhancement compared to bare TiO_2_ nanosheet arrays at the same applied bias potential. This is mainly due to the fact that SnS_2_ outlayer and CoO_x_ catalysts would effectively extend the optical absorption range, accelerate the effective transfer of charge carriers and reduce the charge carrier recombination, thus enhanced photocurrent density. In order to further study the interface charge transport process of photoanodes, electrochemical impedance spectrum (EIS) investigations of the TiO_2_, TiO_2_/SnS_2_, and TiO_2_/SnS_2_/CoO_x_ nanosheet arrays are shown in Fig. 4d, measured at open circuit potential under light illumination (100 mW/cm^2^). Here, *R*_*s*_ denotes the contact resistances of the electrochemical device, CPE denotes the capacitance phase element, and *R*_ct_ denotes the interfacial charge transfer resistance. The values of *R*_ct_ are calculated to be 3780, 2460, and 1650 Ω for TiO_2_, TiO_2_/SnS_2_, and TiO_2_/SnS_2_/CoO_x_ nanosheet array electrodes, respectively. Clearly, a smaller arc radius was observed for TiO_2_/SnS_2_/CoO_x_ as compared to those of TiO_2_ and TiO_2_/SnS_2_ hybrid photoelectrodes. It is noteworthy that the reduction of Nyquist arc radius reflects that an effective separation and fast charge transfer of photoinduced charge carriers have occurred at the hetero-junction interface. These results significantly indicate that the introduction of SnS_2_ and CoO_x_ obviously improve the TiO_2_ PEC properties.

**Fig. 5:**
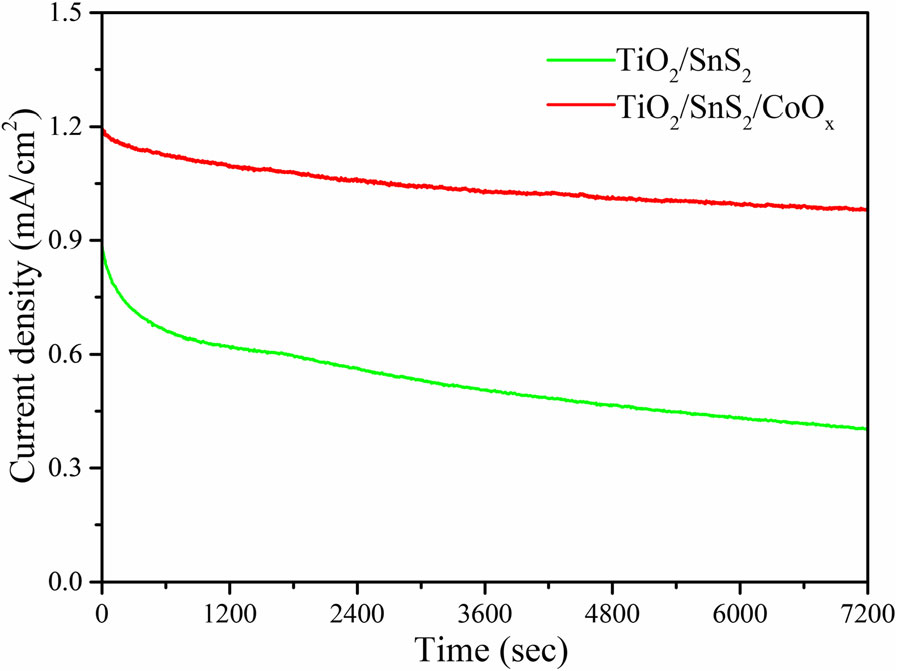
Steady-state photocurrent density curves of the TiO_2_/SnS_2_ and TiO_2_/SnS_2_/CoO_x_ photoelectrodes measured at 1.23 V vs. RHE

On the other hand, the photocurrent stability is also very important to further confirm the PEC performance of water splitting. In order to show the photostability of these photoelectrodes, the long-term stability photostability measurements for TiO_2_/SnS_2_ and TiO_2_/SnS_2_/CoO_x_ nanosheet arrays were carried out for 2 h under the continuous simulated sunlight illumination. As presented in Fig. 5, the decrease in photocurrent density of TiO_2_/SnS_2_ and TiO_2_/SnS_2_/CoO_x_ nanosheet array photoanode is about 54.0% and 18.3% in the following measurement period, respectively. The achieved good stability indicates that the photocorrosion process was restrained after the decoration of CoO_x_ catalysts, and TiO_2_/SnS_2_/CoO_x_ nanosheet arrays still retain the primitive structure under simulated sunlight illumination after long-term PEC water splitting process.

**Fig. 6:**
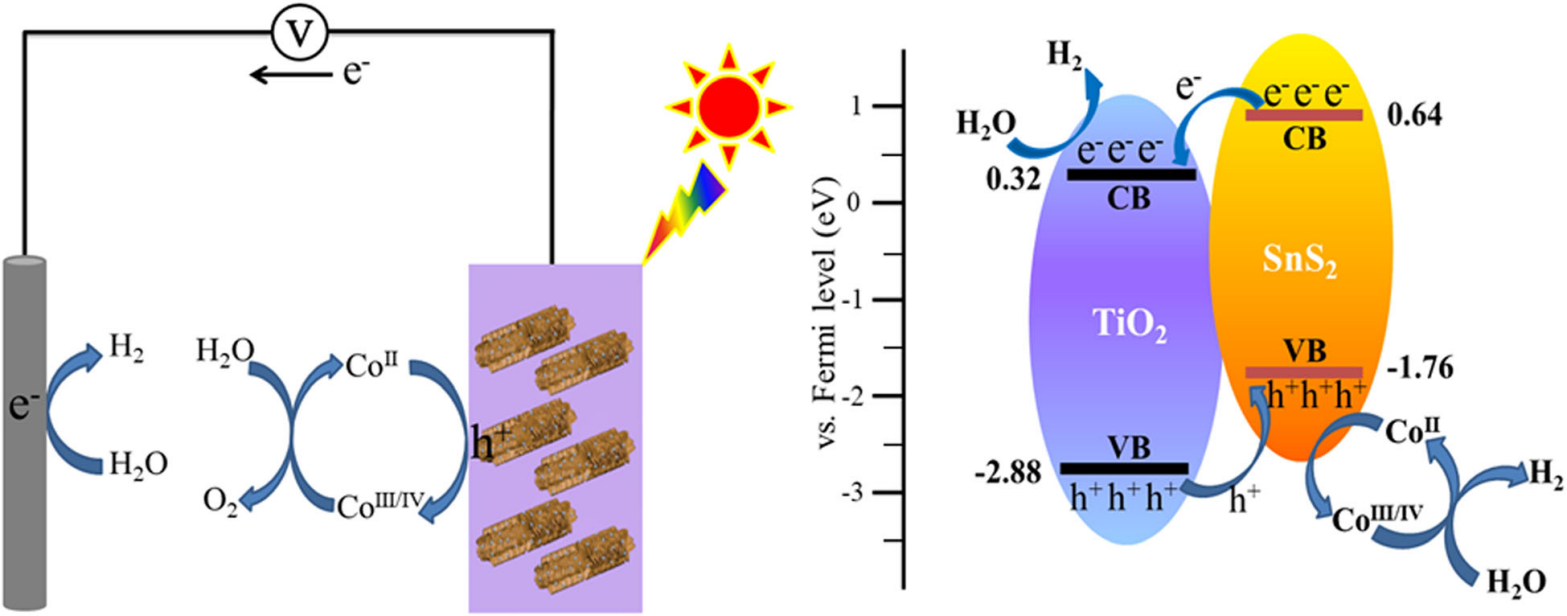
Schematic illustration of device configuration and proposed energy band structure mechanism of TiO_2_/SnS_2_/CoO_x_ photoelectrode

Based on the above results, a possible charge transfer mechanism for the hybrid TiO_2_/SnS_2_/CoO_x_ nanosheet array photoelectrode is proposed in Fig. 6. When the hybrid heterojunction is irradiated by sunlight, as a narrow photosensitizer with excellently high absorption, SnS_2_ is readily excited to generate photoinduced charge carriers under illumination. Eventually, photoinduced electrons on the conduction band (CB) of SnS_2_ can be efficiently transferred to the CB of TiO_2_ nanosheets by the use of the type-II band alignment (Additional file 1: Figure S5), subsequently transmitted to counter electrode through the extra circuit to drive water splitting reactions. Simultaneously, photogenerated holes are transported to the opposite direction from the valence band (VB) of TiO_2_ to VB of SnS_2_ and finally, the photogenerated holes are consumed on the surface of photoanode by photooxidation water process. Furthermore, CoO_x_ nanoparticles couple effectively to the surface layer of hybrid photoanode, which led to the greatly improved photoconversion efficiency under simulated sunlight irradiation. This suggests that CoO_x_ nanoparticles further accelerate photooxidation kinetics, reduce significantly the recombination of photogenerated charge carriers, and restrain photocorrosion of the photoanode, which result in increased PEC performance for water splitting.

## Conclusions

In summary, we have successfully fabricated a novel 2D architecture heterojunction TiO_2_/SnS_2_/CoO_x_ photoanode for PEC water splitting. This ternary hybrid TiO_2_/SnS_2_/CoO_x_ photoanode exhibits significantly enhanced photocurrent density. The photoconversion efficiency of TiO_2_/SnS_2_/CoO_x_ is about 1.8 and 3.6 times higher than that of the TiO_2_/SnS_2_ and pristine TiO_2_ photoelectrodes, respectively. The enhanced PEC performance can be attributable to improve light absorption ability and reduce photo-generated carrier recombination as a result of the type-II heterojunction constructed between TiO_2_ nanosheet and layered SnS_2_. Furthermore, CoO_x_ catalysts further accelerate surface water oxidation kinetics, promote efficient charge separation, and improve PEC stability. This work provides new insight and potential construct of efficient PEC practical applications toward sustainable solar-driven water splitting systems.

## Supplementary information

**Additional file 1: Scheme S1.** A schematic illustration of the formation process for the TiO_2_/SnS_2_/CoO_x_ nanosheet arrays on FTO substrates**. Figure S1.** Cross-sectional SEM images of (a) pristine TiO_2_, (b) TiO_2_/SnS_2_ and (c) TiO_2_/SnS_2_/CoO_x_ nanosheet arrays on FTO substrates, respectively. **Figure S2**. EDS pattern of the TiO_2_/SnS_2_/CoO_x_ nanosheet arrays. **Figure S3.** Raman spectra of pristine TiO_2_ and TiO_2_/SnS_2_ nanosheet arrays. **Figure S4.** Optical bandgap of (a) bare TiO_2_ nanosheet arrays and (b) pristine SnS_2_ samples calculated from the Kubelka-Munk equation. **Figure S5.** (a) UPS spectra of SnS_2_ and (b) XPS valence band spectra of the TiO_2_/SnS_2_ photoelectrode. (DOCX 23095 kb)

## Data Availability

The datasets used or analyzed during the current study are available from the corresponding author on reasonable request.

## Abbreviations

CB: Conduction band
EDS: Energy dispersive X-ray spectrometer
EIS: Electrochemical impedance spectroscopy
FTO: Fluorine-doped tin oxide
LSV: Linear sweep voltammetry
PEC: Photoelectrochemical
RHE: Reversible hydrogen electrode
SEM: Scanning electron microscope
SnS_2_: Tin disulfide
TEM: Transmission electron microscopy
TiO_2_: Titanium dioxide
VB: Valence band
XPS: X-ray photoelectron spectroscopy
XRD: X-ray diffraction

## References

[1] Ma Y, Wang X, Jia Y, Chen X, Han H, Li C (2014). Titanium dioxide-based nanomaterials for photocatalytic fuel generations. Chem Rev.

[2] Yao T, An X, Han H, Chen JQ, Li C (2018). Photoelectrocatalytic materials for solar water splitting. Adv Energy Mater.

[3] Ge M, Cao C, Huang J, Li S, Chen Z, Zhang K-Q, Al-Deyab SS, Lai Y (2016). A review of one-dimensional TiO_2_ nanostructured materials for environmental and energy applications. J Mater Chem A.

[4] Li Y, Wang L, Liang J, Gao F, Yin K, Dai P (2017). Hierarchical heterostructure of ZnO@TiO_2_ hollow spheres for highly efficient photocatalytic hydrogen evolution. Nanoscale Res Lett.

[5] Fujishima A, Honda K (1972). Electrochemical photolysis of water at a semiconductor electrode. Nature.

[6] Schneider J, Matsuoka M, Takeuchi M, Zhang J, Horiuchi Y, Anpo M, Bahnemann DW (2014). Understanding TiO_2_ photocatalysis: mechanisms and materials. Chem Rev.

[7] Cao Z, Yin Y, Yang W, Zhao G, Liu Y, Zhou Y, Peng Y, Wang W, Zhou W, Tang D (2019). Amorphous Co-Pi anchored on CdSe/TiO_2_ nanowire arrays for efficient photoelectrochemical hydrogen production. J Mater Sci.

[8] Zhong D, Jiang Q, Huang B, Zhang W-H, Li C (2015). Synthesis and characterization of anatase TiO_2_ nanosheet arrays on FTO substrate. J Energy Chem.

[9] Yuan Y-J, Ye Z-J, Lu H-W, Hu B, Li Y-H, Chen D-Q, Zhong J-S, Yu Z-T, Zou Z-G (2015). Constructing anatase TiO_2_ nanosheets with exposed (001) facets/layered MoS_2_ two-dimensional nanojunctions for enhanced solar hydrogen generation. ACS Catal.

[10] Gao C, Wei T, Zhang Y, Song X, Huan Y, Liu H, Zhao M, Yu J, Chen X (2019). A photoresponsive rutile TiO_2_ heterojunction with enhanced electron-hole separation for high-performance hydrogen evolution. Adv Mater.

[11] Liu C, Wang L, Tang Y, Luo S, Liu Y, Zhang S, Zeng Y, Xu Y (2015). Vertical single or few-layer MoS_2_ nanosheets rooting into TiO_2_ nanofibers for highly efficient photocatalytic hydrogen evolution. Appl Catal B Environ.

[12] Yu J, Qi L, Jaroniec M (2010). Hydrogen production by photocatalytic water splitting over Pt/TiO_2_ nanosheets with exposed (001) facets. J Phys Chem C.

[13] Yang HG, Liu G, Qiao SZ, Sun CH, Jin YG, Smith SC, Zou J, Cheng HM, Lu GQ (2009). Solvothermal synthesis and photoreactivity of anatase TiO_2_ nanosheets with dominant {001} facets. J Am Chem Soc.

[14] Yang Y, Liu G, Irvine JT, Cheng HM (2016). Enhanced photocatalytic H_2_ production in core-shell engineered rutile TiO_2_. Adv Mater.

[15] Wang L, Li Y, Liu Y (2017). Reduced graphene oxide@TiO_2_ nanorod@reduced graphene oxide hybrid nanostructures for photoelectrochemical hydrogen production. Micro Nano Lett.

[16] Liao J-Y, Lei B-X, Chen H-Y, Kuang D-B, Su C-Y (2012). Oriented hierarchical single crystalline anatase TiO_2_ nanowire arrays on Ti-foil substrate for efficient flexible dye-sensitized solar cells. Energy Environ Sci.

[17] Liu B, Aydil ES (2009). Growth of oriented single-crystalline rutile TiO_2_ nanorods on transparent conducting substrates for dye-sensitized solar cells. J Am Chem Soc.

[18] Bavykin DV, Parmon VN, Lapkin AA, Walsh FC (2004). The effect of hydrothermal conditions on the mesoporous structure of TiO_2_ nanotubes. J Mater Chem.

[19] Sarkar D, Chattopadhyay KK (2014). Branch density-controlled synthesis of hierarchical TiO_2_ nanobelt and tunable three-step electron transfer for enhanced photocatalytic property. ACS Appl Mater Inter.

[20] Zhang Z, Shao C, Li X, Sun Y, Zhang M, Mu J, Zhang P, Guo Z, Liu Y (2013). Hierarchical assembly of ultrathin hexagonal SnS_2_ nanosheets onto electrospun TiO_2_ nanofibers: enhanced photocatalytic activity based on photoinduced interfacial charge transfer. Nanoscale.

[21] Chen HM, Chen CK, Liu RS, Zhang L, Zhang J, Wilkinson DP (2012). Nano-architecture and material designs for water splitting photoelectrodes. Chem Soc Rev.

[22] Liu T, Wang J, Liu L, Feng S, Su P, Yang H, Fu W (2018). Enhanced photoelectric performance of CdS/CdSe co-sensitized TiO_2_ nanosheets array film. Sustainable Energy Fuels.

[23] Wang L, Duan X, Wang G, Liu C, Luo S, Zhang S, Zeng Y, Xu Y, Liu Y, Duan X (2016). Omnidirectional enhancement of photocatalytic hydrogen evolution over hierarchical “cauline leaf” nanoarchitectures. Appl Catal B Environ.

[24] Liu S, Yu J, Jaroniec M (2011). Anatase TiO_2_ with dominant high-energy {001} facets: synthesis, properties, and applications. Chem Mater.

[25] Xiang Q, Yu J, Wang W, Jaroniec M (2011). Nitrogen self-doped nanosized TiO_2_ sheets with exposed {001} facets for enhanced visible-light photocatalytic activity. Chem Commun.

[26] Sajan CP, Wageh S, Al-Ghamdi AA, Yu J, Cao S (2015). TiO_2_ nanosheets with exposed {001} facets for photocatalytic applications. Nano Res.

[27] Han X, Kuang Q, Jin M, Xie Z, Zheng L (2009). Synthesis of titania nanosheets with a high percentage of exposed (001) facets and related photocatalytic properties. J Am Chem Soc.

[28] Li D, Jia J, Zheng T, Cheng X, Yu X (2016). Construction and characterization of visible light active Pd nano-crystallite decorated and C-N-S-co-doped TiO2 nanosheet array photoelectrode for enhanced photocatalytic degradation of acetylsalicylic acid. Appl Catal B Environ.

[29] Banerjee B, Amoli V, Maurya A, Sinha AK, Bhaumik A (2015). Green synthesis of Pt-doped TiO_2_ nanocrystals with exposed (001) facets and mesoscopic void space for photo-splitting of water under solar irradiation. Nanoscale.

[30] Aslam U, Rao VG, Chavez S, Linic S (2018). Catalytic conversion of solar to chemical energy on plasmonic metal nanostructures. Nat Catal.

[31] Zhang Y, He S, Guo W, Hu Y, Huang J, Mulcahy JR, Wei WD (2018). Surface-plasmon-driven hot electron photochemistry. Chem Rev.

[32] Long J, Chang H, Gu Q, Xu J, Fan L, Wang S, Zhou Y, Wei W, Huang L, Wang X, Liu P, Huang W (2014). Gold-plasmon enhanced solar-to-hydrogen conversion on the {001} facets of anatase TiO_2_ nanosheets. Energy Environ Sci.

[33] Liu Y, Chen P, Chen Y, Lu H, Wang J, Yang Z, Lu Z, Li M, Fang L (2016). In situ ion-exchange synthesis of SnS_2_/g-C_3_N_4_ nanosheets heterojunction for enhancing photocatalytic activity. RSC Adv.

[34] Yang C, Wang W, Shan Z, Huang F (2009). Preparation and photocatalytic activity of high-efficiency visible-light-responsive photocatalyst SnS_x_/TiO_2_. J Solid State Chem.

[35] Liu Q, Lu H, Shi Z, Wu F, Guo J, Deng K, Li L (2014). 2D ZnIn_2_S_4_ nanosheet/1D TiO_2_ nanorod heterostructure arrays for improved photoelectrochemical water splitting. ACS Appl Mater Inter.

[36] Li X, Yu J, Low J, Fang Y, Xiao J, Chen X (2015). Engineering heterogeneous semiconductors for solar water splitting. J Mater Chem A.

[37] Su T, Shao Q, Qin Z, Guo Z, Wu Z (2018). Role of interfaces in two-dimensional photocatalyst for water splitting. ACS Catal.

[38] Liu S, Li H, Mo R, Chen Q, Yang S, Zhong J (2016). ZnSe sensitized and co-pi catalyzed TiO_2_ nanowire array photoanode for solar-driven water splitting. J Electrochem Soc.

[39] Wang L, Liu X, Luo J, Duan X, Crittenden J, Liu C, Zhang S, Pei Y, Zeng Y, Duan X (2017). Active site self-optimization by irreversible phase transition of 1T-MoS_2_ in photocatalytic hydrogen evolution. Angew Chem.

[40] Yu J, Xu CY, Ma FX, Hu SP, Zhang YW, Zhen L (2014). Monodisperse SnS_2_ nanosheets for high-performance photocatalytic hydrogen generation. ACS Appl Mater Inter.

[41] Fu Y, Cao F, Wu F, Diao Z, Chen J, Shen S, Li L (2018). Phase-modulated band alignment in CdS nanorod/SnS_x_ nanosheet hierarchical heterojunctions toward efficient water splitting. Adv Funct Mater.

[42] Huang E, Yao X, Wang W, Wu G, Guan N, Li L (2017). SnS_2_ nanoplates with specific facets exposed for enhanced visible-light-driven photocatalysis. ChemPhotoChem.

[43] Luo B, Liu G, Wang L (2016). Recent advances in 2D materials for photocatalysis. Nanoscale.

[44] Yan X, Ye K, Zhang T, Xue C, Zhang D, Ma C, Wei J, Yang G (2017). Formation of three-dimensionally ordered macroporous TiO_2_@nanosheet SnS_2_ heterojunctions for exceptional visible-light driven photocatalytic activity. New J Chem.

[45] Christoforidis KC, Sengele A, Keller V, Keller N (2015). Single-step synthesis of SnS_2_ nanosheet-decorated TiO_2_ anatase nanofibers as efficient photocatalysts for the degradation of gas-phase diethylsulfide. ACS Appl Mater Inter.

[46] Sun X, Yang X, Xiang H, Mi H, Zhang P, Ren X, Li Y, Li X (2019). Nitrogen-doped CoO_x_/carbon nanotubes derived by plasma-enhanced atomic layer deposition: efficient bifunctional electrocatalyst for oxygen reduction and evolution reactions. Electrochim Acta.

[47] Yu J, Zhong Y, Wu X, Sunarso J, Ni M, Zhou W, Shao Z (2018). Bifunctionality from synergy: CoP nanoparticles embedded in amorphous CoO_x_ nanoplates with heterostructures for highly efficient water electrolysis. Adv Sci.

[48] Peerakiatkhajohn P, Yun JH, Chen H, Lyu M, Butburee T, Wang L (2016). Stable hematite nanosheet photoanodes for enhanced photoelectrochemical water splitting. Adv Mater.

[49] Yang L, Chu D, Chen Y, Wang W, Zhang Q, Yang J, Zhang M, Cheng Y, Zhu K, Lv J, He G, Sun Z (2016). Photoelectrochemical properties of Ag/TiO_2_ electrodes constructed using vertically oriented two-dimensional TiO_2_ nanosheet array films. J Electrochem Soc.

[50] Dong Y, He K, Yin L, Zhang A (2007). A facile route to controlled synthesis of Co_3_O_4_ nanoparticles and their environmental catalytic properties. Nanotechnology.

[51] Gabe A, García-Aguilar J, Berenguer-Murcia Á, Morallón E, Cazorla-Amorós D (2017). Key factors improving oxygen reduction reaction activity in cobalt nanoparticles modified carbon nanotubes. Appl Catal B Environ.

[52] Tian F, Zhang Y, Zhang J, Pan C (2012). Raman spectroscopy: a new approach to measure the percentage of anatase TiO_2_ exposed (001) facets. J Phys Chem C.

[53] Tu F, Xu X, Wang P, Si L, Zhou X, Bao J (2017). A few-layer SnS_2_/reduced graphene oxide sandwich hybrid for efficient sodium storage. J Phys Chem C.

[54] Yang L, Zhang Q, Wang W, Ma S, Zhang M, Lv J, He G, Sun Z (2016). Tuning the photoelectronic and photocatalytic properties of single-crystalline TiO_2_ nanosheet array films with dominant {001} facets by controlling the hydrochloric acid concentration. J Mater Sci.

[55] Zhuang HL, Hennig RG (2013). Theoretical perspective of photocatalytic properties of single-layer SnS_2_. Phys Rev B.

[56] Johny J, Sepulveda Guzman S, Krishnan B, Avellaneda Avellaneda D, Shaji S (2018). Nanostructured SnS_2_ thin films from laser ablated nanocolloids: structure, morphology, optoelectronic and electrochemical properties. ChemPhysChem.

[57] Fu CF, Wu X, Yang J (2018). Material design for photocatalytic water splitting from a theoretical perspective. Adv Mater.

[58] Chen H, Lyu M, Zhang M, Feron K, Searles DJ, Dargusch M, Yao X, Wang L (2017). Switched photocurrent on tin sulfide-based nanoplate photoelectrodes. ChemSusChem.

[59] Zhang Z, Huang J, Zhang M, Yuan Q, Dong B (2015). Ultrathin hexagonal SnS_2_ nanosheets coupled with g-C_3_N_4_ nanosheets as 2D/2D heterojunction photocatalysts toward high photocatalytic activity. Appl Catal B Environ.

[60] Wu Z, Xue Y, Zhang Y, Li J, Chen T (2015). SnS_2_ nanosheet-based microstructures with high adsorption capabilities and visible light photocatalytic activities. RSC Adv.

