# Supplementary material for: TiO2 Nanosheet Arrays with Layered SnS2 and CoOx Nanoparticles for Efficient Photoelectrochemical Water Splitting
Source: Nanoscale Res Lett. 2019 Nov 11;14:342. doi: 10.1186/s11671-019-3168-7 (PMC6848439; doi:10.1186/s11671-019-3168-7)
Supplement: Supplementary file 1 — Additional file 1: Scheme S1. A schematic illustration of the formation process for the TiO2/SnS2/CoOx nanosheet arrays on FTO substrates. Figure S1. Cross-sectional SEM images of (a) pristine TiO2, (b) TiO2/SnS2 and (c) TiO2/SnS2/CoOx nanosheet arrays on FTO substrates, respectively. Figure S2. EDS pattern of the TiO2/SnS2/CoOx nanosheet arrays. Figure S3. Raman spectra of pristine TiO2 and TiO2/SnS2 nanosheet arrays. Figure S4. Optical bandgap of (a) bare TiO2 nanosheet arrays and (b) pristine SnS2 samples calculated from the Kubelka-Munk equation. Figure S5. (a) UPS spectra of SnS2 and (b) XPS valence band spectra of the TiO2/SnS2 photoelectrode. (DOCX 23095 kb) [file 11671_2019_3168_MOESM1_ESM.docx]

Supplementary Material

TiO_2_ nanosheet arrays with layered SnS_2_ and CoO_x_ nanoparticles **for efficient photoelectrochemical water splitting**

**Zhou Cao, Yanling Yin, Peng Fu, Dong Li, Yulan Zhou, Yuanwen Deng, Yuehua Peng, Weike Wang, Weichang Zhou, Dongsheng Tang^[[1]](#footnote-1)^**

Synergetic Innovation Center for Quantum Effects and Application, Key Laboratory of Low-dimensional Quantum Structures and Quantum Control of Ministry of Education, School of Physics and Electronics, Hunan Normal University, Changsha, 410081, People’s Republic of China.

Electronic mail: dstang@hunnu.edu.cn


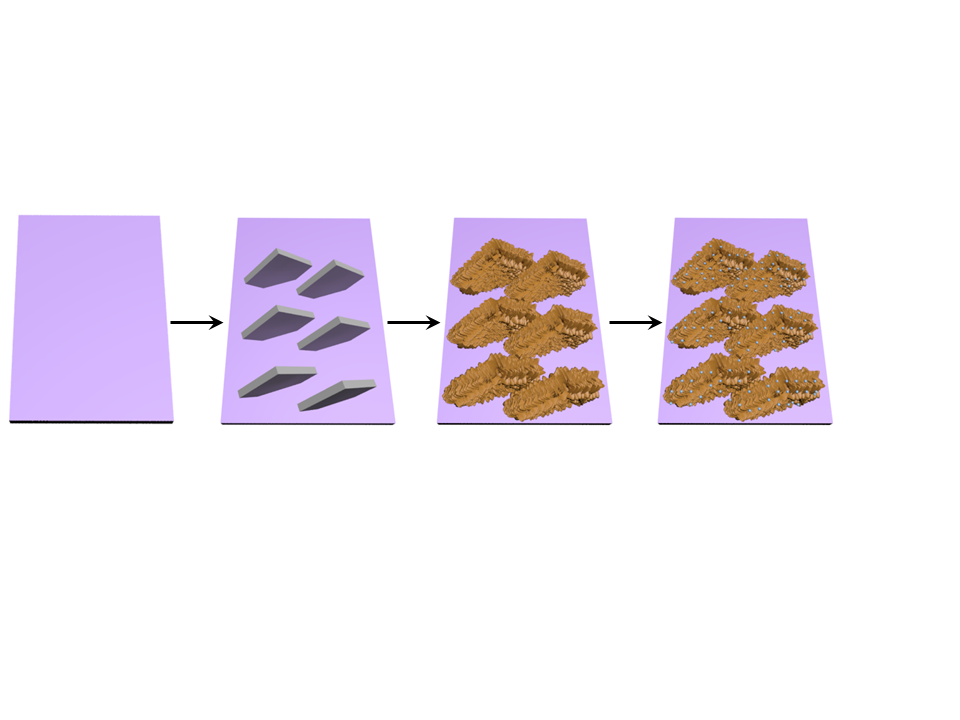


Scheme S1 A schematic illustration of the formation process for the TiO_2_/SnS_2_/CoO_x_ nanosheet arrays on FTO substrates.





Figure S1 Cross-sectional SEM images of (a) pristine TiO_2_, (b) TiO_2_/SnS_2_ and (c) TiO_2_/SnS_2_/CoO_x_ nanosheet arrays on FTO substrates, respectively.





Figure S2 EDS pattern of the TiO_2_/SnS_2_/CoO_x_ nanosheet arrays.





Figure S3 Raman spectra of pristine TiO_2_ and TiO_2_/SnS_2_ nanosheet arrays.





Figure S4 Optical bandgap of (a) bare TiO_2_ nanosheet arrays and (b) pristine SnS_2_ samples calculated from the Kubelka-Munk equation.





Figure S5 (a) UPS spectra of SnS_2_ and (b) XPS valence band spectra of the TiO_2_/SnS_2_ photoelectrode.

1. Author to whom correspondence should be addressed. [↑](#footnote-ref-1)
